# Iminosugar antivirals: the therapeutic sweet spot

**DOI:** 10.1042/BST20160182

**Published:** 2017-04-13

**Authors:** Dominic S. Alonzi, Kathryn A. Scott, Raymond A. Dwek, Nicole Zitzmann

**Affiliations:** Department of Biochemistry, Oxford Glycobiology Institute, University of Oxford, South Parks Road, Oxford OX1 3QU, U.K.

**Keywords:** calnexin, drug discovery and design, glucosidase, glycobiology, iminosugar

## Abstract

Many viruses require the host endoplasmic reticulum protein-folding machinery in order to correctly fold one or more of their glycoproteins. Iminosugars with glucose stereochemistry target the glucosidases which are key for entry into the glycoprotein folding cycle. Viral glycoproteins are thus prevented from interacting with the protein-folding machinery leading to misfolding and an antiviral effect against a wide range of different viral families. As iminosugars target host enzymes, they should be refractory to mutations in the virus. Iminosugars therefore have great potential for development as broad-spectrum antiviral therapeutics. We outline the mechanism giving rise to the antiviral activity of iminosugars, the current progress in the development of iminosugar antivirals and future prospects for this field.

## Introduction

Enveloped viruses have a membrane surrounding their capsid, which is composed of lipids derived from host membranes and viral glycoproteins. These viral glycoproteins are typically dependent upon the host endoplasmic reticulum (ER) protein-folding machinery to form the three-dimensional structure necessary for secretion and/or activity [1–3]. Partial inhibition of the ER-folding mechanisms in order to prevent properly folded viral glycoproteins from being incorporated into the virus is therefore a promising target for the development of broad-spectrum antivirals [4,5].

That effective treatments for viral diseases are an urgent requirement has been highlighted by the recent outbreaks of Ebola and Zika. Whilst vaccines are considered the gold standard in this area, the production of effective vaccines can in some cases take many years and factors such as the presence of multiple viral serotypes, as seen for dengue virus (DENV), can pose an added challenge for vaccine development [6]. A complementary approach to vaccination is the development of antiviral drugs. Antiviral drugs fall into two classes: direct-acting drugs, which target one of the essential components of the virus itself, and host-directed drugs, which target a host process required for viral replication. The development of host-directed antivirals is challenging due to the need to ensure selective toxicity towards the virus; however, they avoid the problem of resistance mutations arising in the viral target, which can limit the efficacy of direct-acting antivirals (see, for example, recent reviews [7,8]).

**Figure 1.**
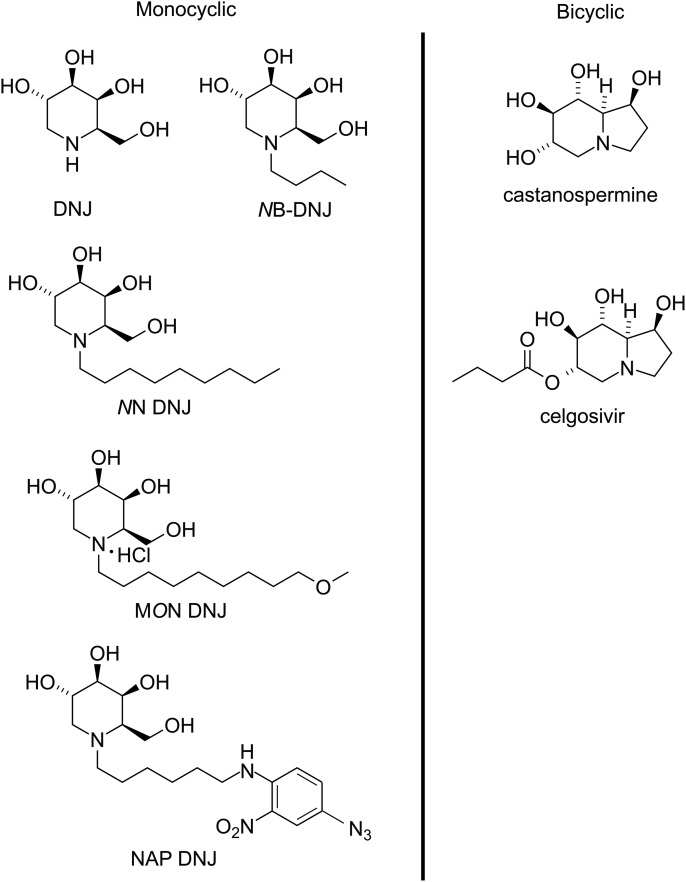
Structures of iminosugars discussed in this mini-review. The left panel shows the structure of the naturally occurring iminosugar DNJ, along with derivatives modified to increase antiviral efficacy. M*O*N-DNJ, currently in clinical trial against dengue, is also referred to as UV4B. The right panel shows the structure of the naturally occurring iminosugar CAST along with the prodrug celgosivir which is in a phase II clinical trial against dengue.

The iminosugar class of compounds includes promising candidates for the development of host-directed antivirals (Figure 1). Iminosugars are sugar mimetics in which the cyclic oxygen is replaced with a nitrogen [9]. Their structural similarity to sugar molecules means that many iminosugars are competitive inhibitors of enzymes that act on sugar substrates. As such, their therapeutic potential has been explored against a range of diseases including Gaucher's disease for which the enzymatic target is glucosylceramide synthase [10,11], and type II diabetes where the targets are intestinal α-glucosidases [12]. Key host enzyme targets for antiviral activity are the ER α-glucosidases I and II (α-glu I and α-glu II), which control entry to the calnexin cycle, which is required for the folding of many glycoproteins [2,13–17]. Iminosugars that inhibit these enzymes have antiviral activity against a range of enveloped viruses including DENV [18,19], influenza virus [20,21], hepatitis C virus (HCV) [22,23] and human immunodeficiency virus [24,25]. Support for the approach of targeting α-glu I in antiviral development is provided by the observations that two patients deficient in this enzyme have no clinical evidence of recurrent viral infections and that cells derived from these patients have a greatly reduced ability to support replication of a variety of different viruses [26]. The α-glu I-deficient patients present with multiple neurologic complications; however, such effects are not expected from antiviral therapy targeting α-glu I as only partial inhibition of the enzyme is necessary for an antiviral effect. In addition, antiviral treatment will not be administered during early development where a lack of α-glu I may be more likely to influence neurologic parameters. The sensitivity of viruses to inhibition of α-glu I and II appears to exceed that of the host proteins, giving a potential therapeutic window in which an antiviral effect occurs without a detrimental effect on the host.

## Mechanism of action

It is widely accepted that a key mechanism by which iminosugars act as antivirals is their ability to disrupt glycoprotein folding, arising from the inhibition of ER α-glu I and II. Early work on haemagglutinin (HA), an influenza envelope glycoprotein, extensively characterised the folding pathway of this protein. Experiments using the bicyclic iminosugar castanospermine (CAST) demonstrated that calnexin was required for efficient folding of HA and that the association with calnexin was dependent on the nature of the glycan [2,15,27,28]. The misfolded states of other viral proteins, such as HIV [29], have also been characterised and direct links observed between iminosugars, misfolding and the antiviral effect. However, in the majority of the cases the evidence for this mechanism of action is less direct; addition of iminosugar leads to impaired virion secretion or secretion of non-infectious virions. Here, we describe the calnexin cycle in more detail and briefly touch on other potential mechanisms of action which may be relevant in some viruses.

**Figure 2.**
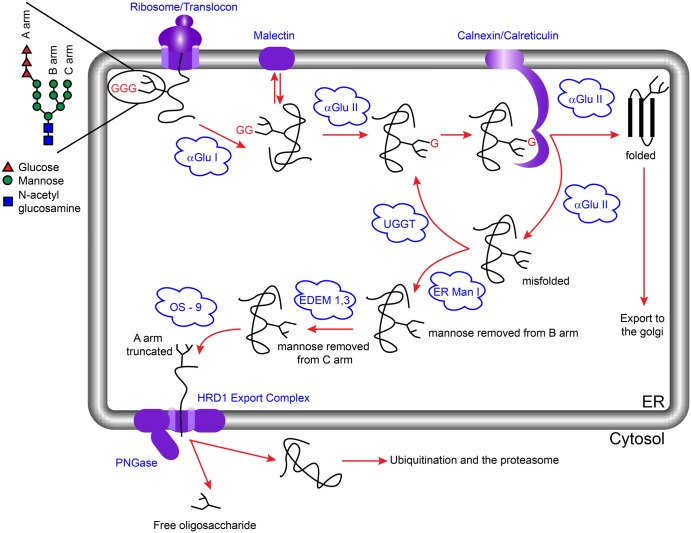
The calnexin cycle and ERAD. The precursor glycan Glc_3_Man_9_GlcNAc_2_ (represented here for simplicity with the glucose residues as red triangles and the remaining portion of the glycan shown as black lines) is added to a peptide co-translationally. Cleavage of the terminal glucose residue by α-glu I leads to a form that can either bind to malectin or be further trimmed by α-glu II to become a substrate for calnexin/calreticulin. On release from calnexin/calreticulin, α-glu II can remove the remaining glucose residue. At this point properly folded proteins are exported to the Golgi for further processing, whilst misfolded proteins are either reglucosylated by UGGT for a ‘second chance’ at folding or directed to the ERAD pathway by ER mannosidase I (ER Man I), which removes a mannose residue from the B-arm of the glycan [42,79]. ER degradation-enhancing α-mannosidase-like proteins 1–3 (EDEM1–3) then act on the C-arm of the glycan followed by OS-9/XTP3-B-mediated delivery of the substrate to the Hrd1 ubiquitination complex through the interaction with a membrane-spanning adaptor protein, SEL1L [80–87]. PNGase separates the glycan from the protein and both segments are degraded [44,88].

N-linked glycoproteins are co-translationally modified at the luminal face of the ER through *en bloc* addition of one or more precursor glycans in the form of Glc_3_Man_9_GlcNAc_2_ by oligosaccharyltransferase [30]. The glycans are recognised and processed by a variety of ER- and Golgi-resident factors that assist protein folding and assembly, mediate flow of secretory cargo and trigger ER-associated degradation (ERAD) [31,32]. Sequential cleavage of the two terminal glucose residues is important for interaction of the nascent polypeptide chain with calnexin, which forms a core part of the ER quality control (ERQC) mechanism [2,15,33,34]. ER α-glu I and α-glu II are the gatekeepers for the calnexin cycle, with binding to ERQC components dependent on the glycoform that the nascent polypeptide retains. ER α-glu I cleaves the terminal glucose residue of the N-linked glycan to give a Glc_2_Man_9_GlcNAc_2_ species. This diglucosylated glycan can be specifically bound by malectin, a membrane-bound ER-resident lectin [35]. Expression of malectin is induced by the unfolded protein response [36], and the protein is proposed to preferentially associate with non-native conformers of folding glycoproteins [37]. The glycan-bound form of malectin potentially associates with the translocon-associated oligosaccharyl transferase acting as an early pathway misfolding sensor [38]. Cleavage of the second glucose residue by α-glu II results in Glc_1_Man_9_GlcNAc_2_, which competes for binding with calnexin/calreticulin and α-glu II [33]. Binding by calnexin retains the protein in the ER where it can interact with chaperones such as binding immunoglobulin protein (BiP) and protein disulfide-isomerase (PDI) [34]. Binding to α-glu II results in cleavage of the third glucose residue after which there are several possible outcomes. If the protein is correctly folded, it can move to the Golgi apparatus for further processing of the glycans. If the protein is misfolded, this may be recognised by UDP-glucose:glycoprotein glucosyl transferase (UGGT), which reglucosylates the glycan such that the protein is once again a substrate for calnexin [39,40]; alternatively, the protein may encounter an α-mannosidase which removes a specific terminal mannose residue targeting the protein for degradation (Figure 2) [41,42].

The presence of large quantities of misfolded proteins will trigger ERAD [32]. This pathway targets misfolded proteins for translocation from the ER into the cytosol, ubiquitination and subsequent hydrolysis by the proteasome. The ERAD targeting presumably occurs through a variety of mechanisms, depending on the nature of the substrate as well as the localisation of the misfolded region within the protein. Glycoproteins degraded through ERAD have their glycan portion released prior to the proteasomal destruction in the cytosol by a peptide:*N*-glycanase (PNGase) [43–45]. As a result, free oligosaccharides (FOS) are produced. These FOS are an excellent biomarker for ERAD and provide both qualitative and quantitative insight into glycoprotein degradation in the ER on a cellular level.

**Figure 3.**
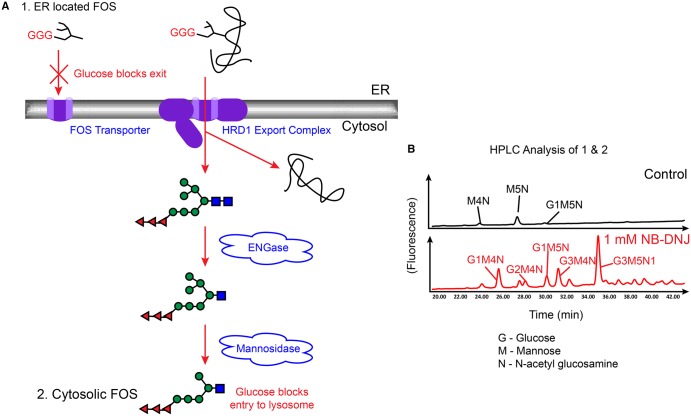
FOS analysis of cells grown in the presence of iminosugars. (**A**) FOS are produced by the activity of two PNGase enzymes: one located in the ER, and the other in the cytosol. In the absence of iminosugar inhibitors FOS produced in the ER will be exported via a FOS transporter to the cytosol for degradation. The presence of terminal glucose residues on the A-arm of the glycan prevents export from the ER, leading to an increase in glucosylated FOS in the ER in the presence of iminosugars. Misfolded glycoproteins targeted for degradation through ERAD are trimmed by the enzymes ENGase and a cytosolic mannosidase. In the absence of iminosugars the resulting glycans enter the lysosome for further degradation; however, the presence of glucose residues prevents this leading to a build-up of glucosylated FOS in the cytosol. (**B**) Following isolation, purification and fluorescent labelling, total cellular FOS containing both the ER pool (1 in A) and cytosolic FOS pool (2 in **A**) can be analysed by NP-HPLC. Shown here are representative chromatograms of total cellular FOS from control HL60s cells (upper panel) and HSL60s cells treated with 1 mM *N*B-DNJ for 24 h (lower panel). The glucose capped glycans visible in the lower panel are indicative of α-glucosidase inhibition.

The FOS produced by PNGase initially carry two GlcNAc residues at the reducing terminus. The cytosolic pathway for FOS catabolism in normal conditions involves rapid processing by endo-β-*N*-acetylglucosaminidase (ENGase), which removes the terminal GlcNAc residue, resulting in a mono-GlcNAc form of FOS [46,47]. This is trimmed by a cytosolic neutral α-mannosidase (NAM, MAN2C1) and subsequently transported into the lysosome for further hydrolysis to monosaccharides [48–50]. Inhibition of α-glu I and II leads to the presence of glucosylated FOS [51,52]. These are processed in the cytosol to the final form of Glc_3_Man_5_GlcNAc_1_ (and slowly to Glc_3_Man_4_GlcNAc_1_; Figure 3). However, glucosylated FOS are unable to access the lysosome, resulting in their cytosolic accumulation [50]. The use of the ER glucosidase iminosugar inhibitor *N*-butyl-deoxynojirimycin (*N*B-DNJ) has demonstrated that the generation of glucosylated FOS is both dose- and treatment time-dependent. Analysing the level of glucosylated FOS in the cytosol is a valuable test for novel iminosugars in a cellular context [53]. This is particularly pertinent, as although there are *in vitro* assays for α-glucosidase inhibition, these do not address the question of cellular uptake. Entry of iminosugars into the ER needs to be achieved and demonstrated for these compounds to be developed for clinical trials.

### Using FOS to probe iminosugar mechanism of action

The calnexin cycle provides a simplistic picture of the current targets involved in inhibiting glycoprotein folding — namely the enzymes α-glu I and α-glu II. The sequential removal of glucose residues, the flux through the ER and the multiple equilibria (between enzymes, inhibitors and competing substrates) involved in the processing of the glycoprotein make it difficult to predict the extent to which each of the two enzymes will be inhibited for any given concentration of iminosugar. We might have expected, for example, that inhibition of α-glu I, which iminosugars such as *N*B-DNJ inhibit at least 10 times more strongly than α-glu II (Table 1), would dominate. However, FOS analysis shows that, at physiologically significant iminosugar concentrations, where an antiviral effect is observed, the result of all the competing reactions and processes is that inhibition of the α-glu II enzyme at the mono-glucosylated stage is key, and that higher concentrations of iminosugars are needed to inhibit predominately α-glu I [53].

The FOS assay carried out on cells incubated with *N*B-DNJ revealed an initial build-up of mono-glucosylated FOS at short treatment periods and low concentrations of iminosugar, before triglucosylated FOS are generated [53]. This reveals the potent cellular inhibition of α-glu II, compared with α-glu I, and contrasts markedly with the inhibition of these enzymes using *in vitro* assays, where, on the basis of IC_50_ values, *N*B-DNJ is 100 times more efficient at inhibiting α-glu I than II. This result is mirrored by more potent α-glu I inhibitors such as NAP-DNJ, which inhibits at low nanomolar concentrations (Table 1) [54]. The very small amount of diglucosylated FOS produced by iminosugar inhibition in the cell demonstrates that iminosugars are relatively poor at preventing the removal of the first α1,2-linked glucose. This reflects the kinetics of α-glu I and II action in the ER, which results in the efficient and rapid hydrolysis of tri- and di-glucosylated glycans to mono-glucosylated glycans, allowing interaction with calnexin/calreticulin chaperones.

The longer half-life of mono-glucosylated glycans, compared with that of tri- and di-glucosylated species, is due to the slower hydrolysis rate of the proximal glucose residue by α-glu II [55]. This contributes to a more favourable environment for iminosugar inhibition of this step. Removal of the first glucose residue by α-glu I and the second glucose residue by α-glu II in cultured cells probably occurs close to their limiting rates (*V*_max_), where addition of a competitive inhibitor has a limited effect on the observed rate. Removal of the third glucose residue by α-glu II is much slower, suggesting that this rate is not close to its *V*_max_, and under such conditions a competitive inhibitor has a greater effect on the rate. The calnexin molecule acts as an anchor, while chaperones, such as BiP and PDI, help with the folding process. Since α-glucosidase II and calnexin both compete for the same substrate in the ER, Glc1Man9GlcNac2-protein, if α-glu II is unable to hydrolyse the substrate bound to calnexin, the presence of calnexin will significantly reduce the free substrate concentration, hence reducing the rate. Alternatively, if α-glu II was able to hydrolyse the substrate bound to calnexin (although such a scenario is difficult to envisage), then the presence of calnexin is likely to change the *K*_m_. An increase in *K*_m_ would also result in a reduced rate. Both possibilities have the same functional outcome: the rate of removal of the proximal glucose residue is reduced in cells and hence is more sensitive to the presence of a competitive inhibitor, resulting in a greater accumulation of mono-glucosylated glycans. The retention of the mono-glucosylated form by inhibition of α-glu II by an iminosugar will result in the glycoprotein failing to pass the quality control and it being targeted for degradation in the ER. It is important to note that not all glycoproteins require the calnexin cycle to fold correctly. The Glc_1_Man_9_GlcNAc_2_ glycan exists in a dynamic equilibrium between two structures, a major and a minor conformer for the Glcα1–3Manα linkage [56]. Modelling of the binding of Glc_1_Man_9_GlcNAc_2_ to calnexin suggests that it is the minor conformer that is recognised by calnexin. This may be one of the mechanisms for controlling the rate of recruitment of proteins into the calnexin/calreticulin chaperone system and enabling proteins that do not use this particular quality control mechanism for folding to bypass the system.

### Inhibition of glycolipid processing

Although it is clear that inhibition of ER α-glu I and/or II has an antiviral effect against many viruses, this may not be the whole story. Monocyclic compounds such as 1-deoxynojirimycin (DNJ) and its derivatives are also inhibitors of glycolipid processing enzymes [52]. Glycolipids play an important role in the life-cycle of many viruses; neutral sphingolipids are thought to be important for the binding of DENV to mammalian and mosquito cell surfaces [57,58], whilst glycosphingolipids are a major component of the lipid rafts necessary for the replication of HCV [59,60]. It is therefore possible that the antiviral effects of iminosugars against these viruses arise from inhibition of glycolipid processing in addition to inhibiting glycoprotein folding. The extent to which inhibition of glycolipid processing contributes to antiviral effects will almost certainly be virus-dependent. Recently, Sayce et al*.* demonstrated that, in the case of DENV, the antiviral effects arise via inhibition of glycoprotein folding and that iminosugar effects on glycolipid processing do not contribute to the antiviral effect [61,62]. For HCV and other viruses the picture is less clear and investigations in this area are ongoing.

## Cellular targets visualised by structural biology

**Figure 4.**
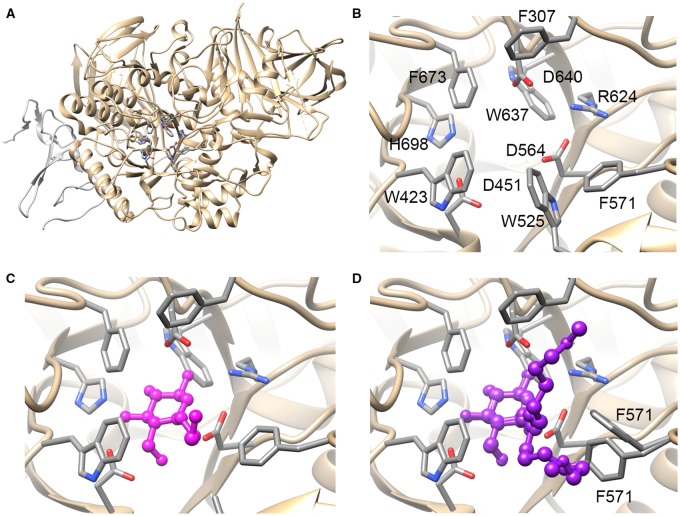
The structure of α-glu II; active site and in complex with inhibitors. (**A**) Ribbon diagram illustrating the structure of α-glu II with the key catalytic residues shown as sticks. The catalytic subunit is shown in gold, whilst the fragment of the accessory subunit remaining after protease digestion is shown in silver. (**B**) The catalytic binding site of α-glu II. Key residues for catalytic activity and interaction with the substrate glycan are shown as sticks. In the catalytic cycle D564 acts as a nucleophile and D640 as the catalytic acid/base. As is typical for sugar-binding sites, there are many aromatic residues and polar groups offering a wide range of potential interactions for inhibitors. (**C**) The catalytic binding site of α-glu II with *N*B-DNJ shown in magenta as a ball-and-stick representation. In this structure, the glucose-like portion occupies a similar position to the natural substrate and the alkyl chain occupies the +1 site, which would be occupied by the sugar moiety adjacent to that cleaved in the reaction in the natural substrate. The side chain of W525 becomes disordered and is not shown in the structure. (**D**) The catalytic binding site of α-glu II with the inhibitor M*O*N-DNJ shown in purple as a ball-and-stick representation. In this structure, the alkyl chain of the iminosugar fully occupies the +1 site and extends beyond; the alkyl chain adopts two main conformations, one of which is located towards the +2 sugar-binding site. Addition of the inhibitor leads to disorder in the catalytic site including displacement of the 523–528 loop (containing W525) and multiple conformations for F571. In all figures, H atoms are removed for clarity and all structures are shown in the same orientation. Protein databank file 5foe.pdb was shown in **A** and **B**; 5ieg.pdb and 5ief.pdb were used for **C** and **D**, respectively.

It is clear that both α-glucosidases are important antiviral targets. Recent structural biology approaches have attempted to examine their structures to both understand the mechanism of inhibition and provide insights into the design and evaluation of more selective and potent inhibitors [63–65]. Inhibition is based on a DNJ structure, which has glucose stereochemistry, and hence, with the abundance of glucose usage in the cell, the chances of multiple binding partners for the drugs are high. Indeed, DNJ compounds with alkyl chains have shown efficacy against enzymes including ER α-glucosidases, gut α-glucosidases, ceramide glucosyl transferase Β and glucocerebrosidases (GBA 1, 2 and 3). The 2.04 Å crystal structure of ER α-glu I determined in 2013 provides clear indication of the catalytic residues, but unfortunately the crystal packing prohibits experimental active-site investigations due to occlusion of the active site by a His_6_ purification tag from a crystal contact [63]. More recently, Caputo et al*.* [64] have solved the structure of mouse α-glu II (*Mm*α-GluII) in the presence of a series of substrate analogues, inhibitors and products (Figure 4). When compared with the structure of an intestinal α-glucosidase, the structure has revealed some crucial differences including an ‘exclusion loop’ unique to α-glu II that hangs over the active site. Generating compounds that bind to the exclusion loop region may therefore lead to increased specificity for ER α-glu II over the intestinal α-glucosidases. In addition, crystal structures with inhibitors gave insight into potential methods for designing inhibitors with increased affinity. Alkylated DNJ derivatives, for example, occupy the site of the terminal glucose residue as expected, but the extent to which they occupy the binding sites for the adjacent sugar moieties depends upon the length of the alkyl chain. *N*B-DNJ, with its four carbon alkyl chain, occupies the +1 sugar-binding site, adjacent to the terminal glucose site, whilst M*O*N-DNJ with its much longer chain extends towards the +2 sugar-binding site. The co-crystal structure with M*O*N-DNJ suggests that the aromatic residues forming a ring between the +1 and +2 site would be good targets for inhibitors with increased potency (Figure 4C,D).

## The consequences of misfolding

Iminosugars show antiviral activity against viruses from a wide range of different families, thus it appears that glycoproteins from many viruses are dependent upon the calnexin cycle for proper folding. There is, however, indirect evidence that the extent of misfolding caused by iminosugar inhibition of α-glucosidases varies between viruses. For some viruses, such as HIV, the level of secreted virus decreases by only a small amount in the presence of iminosugars, but the secreted viral particles have greatly reduced infectivity [29]. This implies that the degree of misfolding is small enough to escape detection by the cellular unfolded protein response but large enough to impair function. In contrast, for other viruses (including DENV, Hepatitis B virus and bovine viral diarrhea virus) the presence of iminosugars leads to greatly reduced secretion of virions [1,19,66]. This suggests that the misfolding of the glycoproteins in this case is more severe. In the case of Hepatitis B, at high doses of iminosugars (where α-glu I is inhibited and viral glycoproteins with the unmodified Glc_3_Man_9_GlcNAc_2_ glycoform will predominate) a considerable fraction of the viral glycoproteins are retained within the cell. These glycoproteins appear as aggregates and have an intracellular half-life in excess of 24 h. It was hypothesised that one particular viral glycoprotein (M) when in the triglucosylated state may act as a kind of ‘poison pill' in the ER preventing the secretion of the virus. In this case a viral protein acts as a ‘dominant negative' poison of viral secretion and may itself be considered the antiviral drug. The long intracellular half-life of the ‘poison pill’ may be due to aberrant trafficking between the ER and Golgi that has recently been observed for glycoproteins retaining terminal glucose residues on their glycans. This additional mechanism could lead to a reservoir of unfolded protein interfering with the ERQC. These are areas that need to be investigated further to understand the structural requirements for viral assembly, secretion and fusion to host cells in an iminosugar protein folding compromised context.

Currently, we cannot yet predict which glycoproteins will be secreted and which degraded in the presence of an iminosugar, and it remains a challenge to identify whether inhibition of ER α-glu I versus α-glu II will affect the extent of misfolding observed.

## Clinical prospects and challenges

Naturally occurring iminosugars including DNJ and CAST have been used as the starting point for medicinal chemistry strategies aimed at antiviral drug discovery. Compounds modified from these starting materials have been, or are currently being, evaluated in clinical trials against HIV, HCV and DENV [67–71].

The *n*-butylated form of DNJ (*N*B-DNJ) and celgosivir, a prodrug of CAST, were both evaluated in clinical trials against HIV; however, neither proceeded further than phase II [67,68]. In the case of *N*B-DNJ, although some effects on viraemia were observed, it proved impossible to maintain therapeutic concentrations of the drug in serum. Observations that the concentrations of *N*B-DNJ necessary to inhibit the ER α-glucosidases in whole-cell assays greatly exceed that necessary to achieve the same inhibition of isolated enzymes suggested that difficulties in penetration of the drug into the ER were responsible for these poor results. These trials did, however, demonstrate that both DNJ and celgosivir are well tolerated by patients. The principal side effect of DNJ-derived iminosugars is osmotic diarrhoea, which arises due to the inhibition of the gut α-glucosidases [68,72]. This side effect is reversible and can be controlled by diet; however, improvements to limit the unwanted effect would be desirable, particularly in the development of iminosugar antivirals to treat chronic viral infections.

The current most promising targets for iminosugar antivirals appear to be DENV, for which clinical trials are in process [70,71], and influenza, where promising results have been observed in preclinical studies [21,73]. DENV is a mosquito-borne disease estimated to infect up to 390 million people per annum [74]. Many of these infections have no clinical manifestation, whilst others require hospitalisation, and a small percentage develop into dengue haemorrhagic fever, which can be fatal [75]. Both celgosivir and the DNJ-derivative UV-4 (M*O*N-DNJ) are currently in clinical trials against DENV. UV-4 is in a phase I clinical trial, with a study evaluating the pharmacokinetics of the hydrochloride salt of UV-4 (UV-4B) administered as a multiple ascending dose [71]. Celgosivir has recently been approved for a phase II clinical trial [70]. In a proof of concept phase 1b study in which 50 patients were recruited, administration of celgosivir did not appear to reduce the viral load or fever burden in patients with uncomplicated dengue fever [76]. However, further investigations using a mouse model suggest that the dosing regime may be critical for efficacy. Mouse studies carried out prior to the phase 1b study suggested that a twice-daily treatment regime would be sufficient to reduce viraemia [77]; however, work carried out in light of the results from the phase 1b clinical trial suggests that this treatment regime is inadequate when the treatment is begun at the peak of viraemia [78]. More recent work suggests that a four-time daily regime is likely to be more effective against established infections and this will be investigated in the upcoming trial. A range of iminosugars, including *N*B-DNJ, *N*N-DNJ and UV-4B, have been tested for efficacy against the influenza virus [20,21,73]. Intriguingly, the antiviral efficacy observed varies between different influenza strains and subtypes in addition to varying with the specific iminosugar inhibitor tested. In a recent study Warfield et al*.* demonstrated that, at high concentrations, UV4-B shows activity against both influenza A and influenza B strains in cell culture, and that it gave a significant survival benefit against influenza A H1N1 and H3N2, and influenza B/Sichuan/379/99 in lethal mouse models. Future studies on the efficacy against influenza in humans are planned [21].

## Conclusions

Our understanding of the mechanism of action of iminosugar antivirals has developed enormously since DNJ was first reported to have an antiviral effect against HIV. Medicinal chemistry approaches have improved the efficacy and pharmacokinetics of natural iminosugars, and current compounds represent good prospects for iminosugar antivirals reaching the clinic. However, medicinal chemists are already leading the way towards the ‘next generation’ of iminosugars. The compounds currently under investigation are well tolerated by patients and offer hope against current and future viral challenges; however, it is always desirable to limit off-target effects and increase ER uptake. The structures of mammalian ER α-glucosidases, together with those of off-target α-glucosidases, have the potential to facilitate rational design of new inhibitors with increased specificity for the ER-resident enzyme(s).[Table BST-2016-0182CTB1]

**Table 1 BST-2016-0182CTB1:** Iminosugar IC_50_ values against purified rat α-glucosidase I and II; experiments were performed using a Glc_1-3_Man_7_GlcNAc_2_ substrate

Iminosugar	α-glu I	α-glu II	
		Glc2	Glc1
*N*B-DNJ	0.68 ± 0.05	10.80 ± 05	53.00 ± 6.6
*N*N-DNJ	0.54 ± 0.08	2.10 ± 0.42	14.90 ± 3.6
M*O*N-DNJ	0.015 ± 0.04	0.90 ± 0.19	1.60 ± 0.2
NAP-DNJ	0.017 ± 0.01	0.30 ± 0.1	0.83 ± 0.13

## Abbreviations

BiP: binding immunoglobulin protein
CAST: castanospermine
DENV: dengue virus
ENGase: endo-β-*N*-acetylglucosaminidase
ER: endoplasmic reticulum
ERAD: ER-associated degradation
ERQC: ER quality control
FOS: free oligosaccharides
HA: haemagglutinin
HCV: hepatitis C virus
MON-DNJ: N-(9′-methoxynonyl)-1-deoxynojirimycin
NAP-DNJ: N-(6′-4″-azido-2″-nitrophenylamino) hexyl-1-deoxynojirimycin
*N*B-DNJ: *N*-butyl-deoxynojirimycin
NN-DNJ: N-nonyl-deoxynojirimycin
PDI: protein disulfide-isomerase
PNGase: peptide:*N*-glycanase
UGGT: UDP-glucose:glycoprotein glucosyl transferase
